# Effects of Periostracum Cicadae on Cytokines and Apoptosis Regulatory Proteins in an IgA Nephropathy Rat Model

**DOI:** 10.3390/ijms19061599

**Published:** 2018-05-29

**Authors:** Lu Yang, Yan Wang, Aobulikasimu Nuerbiye, Ping Cheng, Jin-Hui Wang, Rena Kasimu, Hong Li

**Affiliations:** 1 Xinjiang Academy of Forestry, Urumqi 830000, China; yanglukitty127@163.com (L.Y.); 18999537981@163.com (Y.W.); chengping1985@163.com (P.C.); 2 Economic Forest Product Quality Inspection and Testing Center of the State Forestry Administration (Urumqi), Urumqi 830052, China; 3 Xinjiang Medical University, Urumqi 830000, China; renakasimu@vip.sina.com or xjlky_2@163.com (A.N.); tcm_shz@aliyun.com (J.-H.W.); renakasimu@vip.sina.com (R.K.); 4 Harbin Medical University, Harbin 150081, China

**Keywords:** periostracum cicadae, IgA nephropathy, inflammation, fibrosis, apoptosis

## Abstract

Periostracum cicadae, the cast-off shell of the cicada *Cryptotympana pustulata* Fabricius, is used in traditional Chinese medicine for its diaphoretic, anticonvulsive, sedative, antipyretic, and antiallergic effects. However, the exact pathogenesis of immunoglobulin A nephropathy (IgAN) remains unclear, thereby hindering investigations to identify novel therapeutic agents. A rat IgAN model was established by administration of bovine serum albumin, lipopolysaccharide, and carbon tetrachloride, which simultaneously established blood stasis and a heat syndrome model. The animals were sacrificed to detect changes in protein levels in urine and blood. Immunofluorescence was performed to assess IgA deposition in the glomeruli. Tumor necrosis factor-α (TNF-α), interleukin-1β (IL-1β), and interleukin 6 (IL-6) levels were measured in bronchoalveolar lavage fluid (BALF) by enzyme-linked immunosorbent assay. Hematology and eosin, periodic acid-Schiff, TUNEL (TdT-mediated dUTP Nick-End Labeling), and immunohistochemical staining were performed to evaluate histopathological changes in kidney tissues. Additionally, target-related proteins were measured by Western blotting. Periostracum cicadae resulted in a reduction in blood and urine protein levels. Serum TNF-α, IL-1β, and IL-6 levels significantly decreased in the periostracum cicadae-treated groups compared to the IgAN group. Furthermore, a reduction in MCP-1 (Monocyte Chemotactic Protein-1), TLR4 ((Toll-Like Receptor 4)), and IgA expression levels and a dose-dependent increase in caspase 3 expression were observed in response to periostracum cicadae treatment. TGF-β1(Transforming Growth Factor-β) levels decreased, whereas that of Fas increased in the kidney tissues of the periostracum cicadae-treated groups. The findings of the present study indicate that periostracum cicadae induces apoptosis and improves kidney inflammation and fibrosis in IgA nephropathy rat models.

## 1. Introduction

Immunoglobulin A nephropathy (IgAN) is the most common type of glomerulonephritis in the world [1]. IgA nephropathy is characterized by immune deposits predominantly composed of polymeric IgA in the glomerular mesangium [2,3,4,5]. Nephrotic-range proteinuria, microscopic hematuria, and renal failure impairment are strong predictors of an adverse clinical outcome [6,7,8]. The risk of progression to chronic kidney disease in patients with IgAN in adults is apparently significantly higher in children. IgAN patients with proteinuria are generally considered to have increased risk for progression to chronic kidney disease (CKD) and renal failure [9]. Renal involvement is the most serious long-term complication; its symptoms include asymptomatic microhematuria and/or mild proteinuria to overt IgAV nephritis (IgAVN) [10]. IgAVN, which occurs in approximately 30% of pediatric patients within 4–6 weeks of initial presentation [11], and severe IgAVN, are both associated with decreased renal function, hypertension, hypoalbuminemia, and long-term renal sequelae. Current treatment for IgAVN, which include steroids and immunosuppressive drugs, are mainly based on the findings of studies on IgAN [12]. Recently, Oxford presented a new classification for IgA nephropathy as proposed by a Working Group of the International IgA Nephropathy Network and the Renal Pathology Society [13], which has been validated by numerous studies [14,15,16,17,18]. This group identified four histopathologic features that are associated with the progression of kidney disease: mesangial hypercellularity score (M; M0 ≤ 0.5, M1 ≥ 0.5), the presence of endocapillary proliferation (E; E0: absent, E1: present), segmental glomerulosclerosis/adhesion (S; S0: absent, S1: present), and the severity of tubular atrophy/interstitial fibrosis (T; T0 ≤ 25%, T1: 26–50%, T2 ≥ 50%). However, the exact pathogenesis of IgAN remains unclear, thereby hindering research investigations to identify novel therapeutic agents for the treatment of this particular disease.

The traditional Chinese crude drug periostracum cicadae is the cast-off shell of *Cryptotympana pustulata* Fabricius, commonly known as the black cicada. Periostracum cicadae has a sweet and salty taste, and produces a “cooling” effect. Periostracum cicadae is known as chantui in traditional Chinese medicine, and has been historically used in the treatment of sore throat, hoarseness, itching, spasms, and other symptoms. The crude extract of periostracum cicadae can be used as a sedative, hypothermic, anticonvulsive [19], antioxidant, anti-inflammatory [19,20], antipyretic, and antiallergic agent [21], as well as a sympathetic ganglionic blocker [22], when administered by various routes. To our knowledge, this is the first study that has investigated the molecular mechanism of periostracum cicadae in inflammation and fibrosis using an IgAN model.

## 2. Results

### 2.1. The Effect of Periostracum Cicadae on Proteinuria and Other Biochemical Parameters

**Figure 1:**
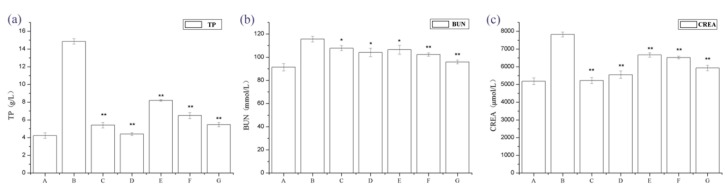
Changes in 24 h urine protein, blood urea nitrogen (BUN), and serum creatinine (Scr) levels. * *p* < 0.05, ** *p* < 0.01 compared to the immunoglobulin A nephropathy (IgAN) model group. A: Normal, healthy controls; B: IgAN rat models; C: prednisone acetate group (PAG); D: tripterygium glycoside tablet group; E–G: treatment group that received 0.5, 1, and 2 g/kg periostracum cicadae, respectively.

We examined the physical appearance and biochemical parameters of the rats. The IgAN rats showed gray hair, slow growth, and a reduced amount of activity. After molding, urine from rats was collected at 24 h, and it was observed that IgAN rats developed macroscopic hematuresis, however, the normal group and treatment group had no expression. The IgAN rat models showed significantly higher urine protein, blood urea nitrogen (BUN), and serum creatinine (Src) levels than the normal, healthy controls, whereas these were markedly lower in the treatment group (Figure 1). These findings indicate that periostracum cicadae treatment lowers protein, BUN, and creatinine (CREA) levels in the IgAN rat model.

**Figure 2:**
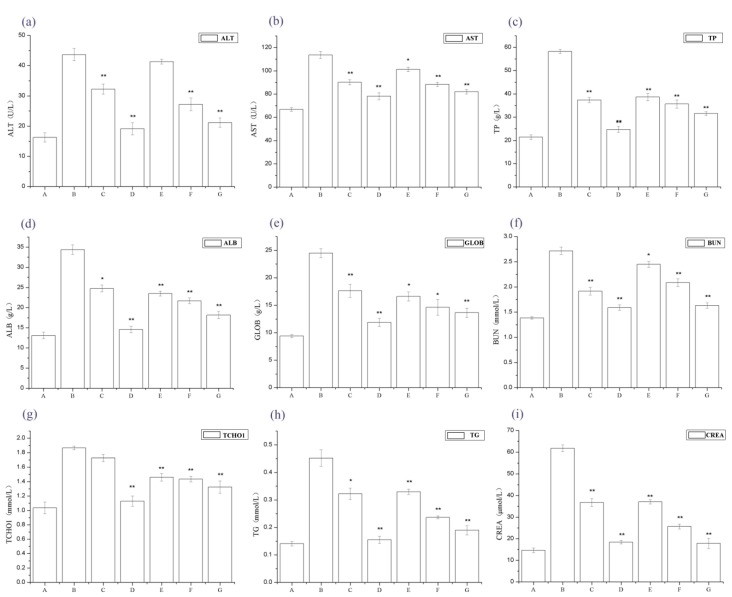
Changes in serum alanine aminotransferase (ALT), aspartate aminotransferase (AST), total protein (TP), total cholesterol (TCHO), triacylglyceride (TAG), blood urea nitrogen (BUN) and creatinine (CREA) levels. * *p* < 0.05, ** *p* < 0.01 compared to the IgAN model group. A: Normal, healthy controls; B: IgAN model group; C: prednisone acetate group; D: tripterygium glycoside tablet group; E–G: treatment group that received 0.5, 1, and 2 g/kg periostracum cicadae, respectively.

Figure 2 shows that serum alanine amino transferase (ALT), aspartate aminotransferase (AST), total protein (TP), Serum albumin (ALB), Globulin (GLOB), BUN, total cholesterol (TCHO1), CREA, and TG levels significantly increased in the IgAN rat models compared to normal, healthy controls, whereas serum ALT, AST, TP, ALB, GLOB, BUN, TCHO1, CREA, and TG levels dramatically decreased in the periostracum cicadae treatment group, compared to the IgAN model group.

### 2.2. The Effect of Periostracum Cicadae on IgA

**Figure 3:**
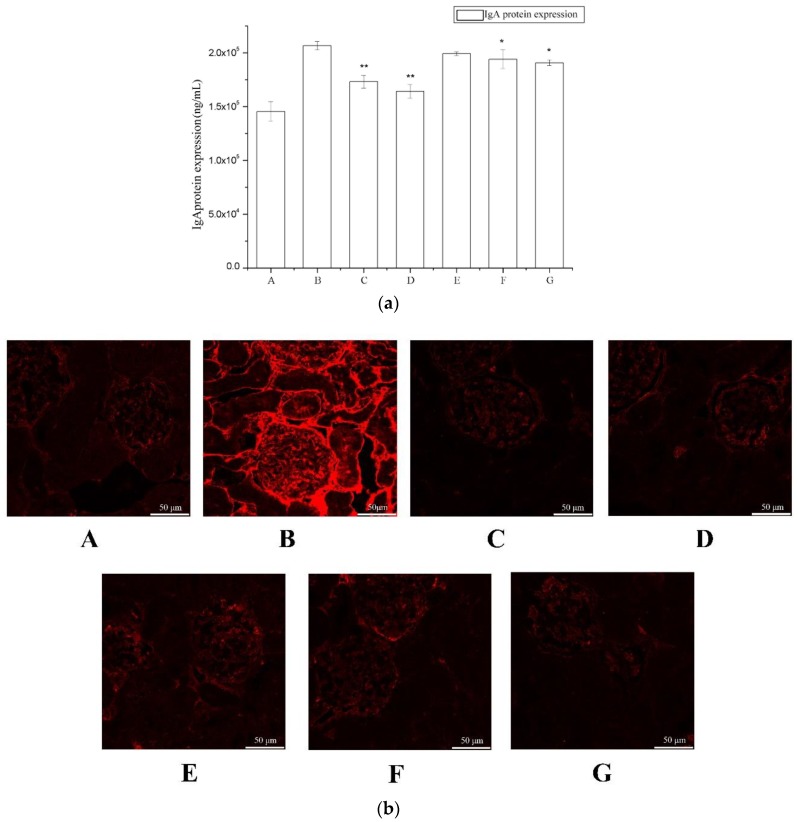
The effect of periostracum cicadae on IgA deposition. (**a**) Effects of periostracum cicadae on serum IgA levels in IgAN rats (* *p* < 0.05, ** *p* < 0.01). (**b**) Immunofluorescence staining of IgA in the glomeruli. A: Normal, healthy controls; B: IgAN model group; C: prednisone acetate group; D: tripterygium glycoside tablet group; E–G: treatment group that received 0.5, 1, and 2 g/kg periostracum cicadae.

To evaluate the effects of periostracum cicadae on IgA levels, we measured serum IgA levels by ELISA (Enzyme Linked Immunosorbent Assay) (Figure 3a). The IgA levels of the model group were significantly higher compared to the untreated control group, whereas these were significantly lower in the treatment group relative to the model group.

IgA deposits in the glomeruli were observed by immunofluorescence staining (Figure 3b). The control rats showed minimal IgA deposition in the glomeruli, with an average fluorescence intensity of “−” (Table 1). In contrast, the IgAN rats exhibited significant mass- or granule-like red fluorescence that was scored as “+” to “++++”; however, these images did not provide evidence for increased deposition in the thick loop region. The majority of the fluorescence was observed in the glomeruli. These findings were indicative that the IgAN animal model was successfully established.

### 2.3. Periostracum Cicadae Alleviates Inflammation in Rats with IgAN

**Figure 4:**
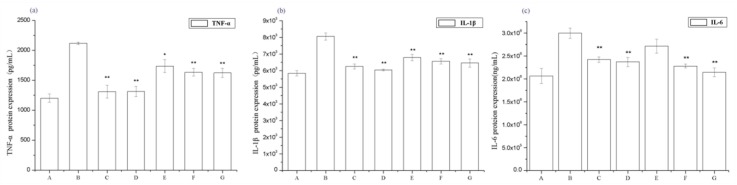
Effects of periostracum cicadae on the serum levels of tumor necrosis factor-α (TNF-α), interleukin-1β (IL-1β), and interleukin 6 (IL-6) in IgAN rats (* *p* < 0.05, ** *p* < 0.01). A: Healthy, normal control group; B: IgAN model group; C: prednisone acetate group; D: tripterygium glycoside tablet group; E–G: treatment group that received 0.5, 1, and 2 g/kg periostracum cicadae, respectively.

To determine the effect of periostracum cicadae on the renal inflammatory responses of rats, the serum levels of the inflammatory cytokines TNF-α, IL-1β, and IL-6 were evaluated. The serum TNF-α, IL-1β, and IL-6 levels of the IgAN model group significantly increased compared to those in the untreated control group, whereas those of the treatment group were significantly lower than the IgAN model group (Figure 4). These data show that periostracum cicadae treatment lowers serum TNF-α, IL-1β, and IL-6 levels, indicating that periostracum cicadae has anti-inflammatory properties that alleviate IgAN-induced kidney fibrosis in rats.

### 2.4. The Effect of Periostracum Cicadae on Inflammatory Cell Infiltration

**Figure 5:**
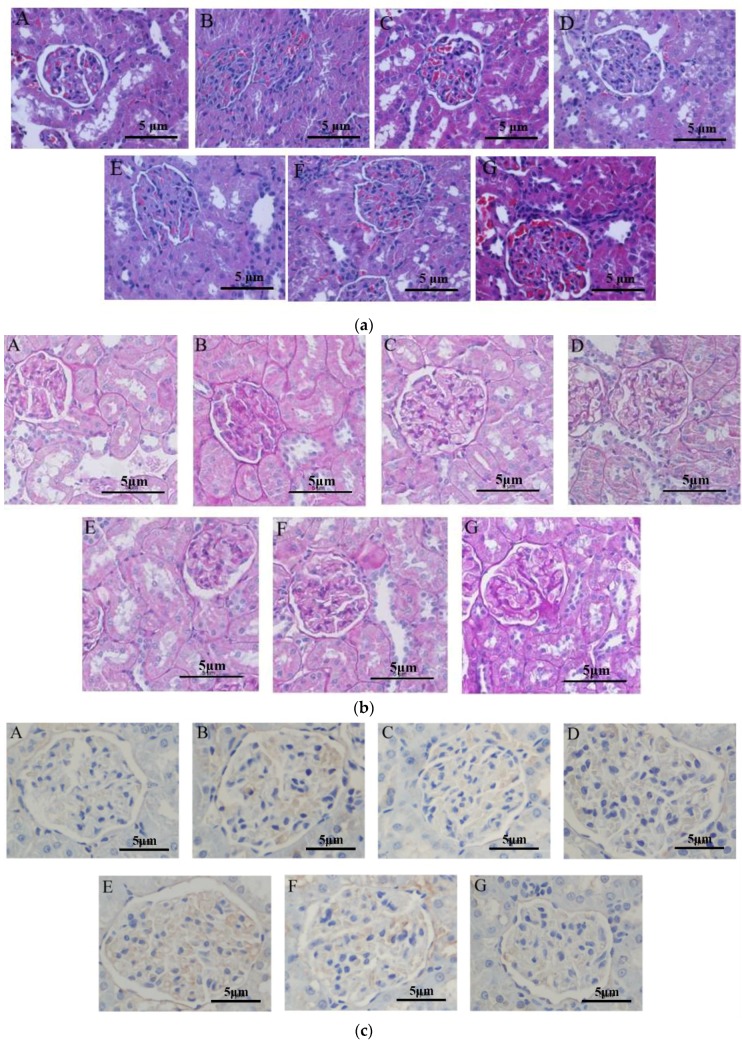
Periostracum cicadae improves kidney inflammation and fibrosis and induces cell apoptosis. (**a**) Hematoxylin and eosin (H&E) staining. (**b**) Periodic acid-Schiff (PAS) staining. (**c**) TdT-mediated dUTP nick end-labeling (TUNEL) staining.

Asthmatic mice that received periostracum cicadae were histopathologically evaluated using hematoxylin and eosin (H&E) (Figure 5a) and periodic acid-Schiff (PAS) (Figure 5b) staining. In the untreated control group (Figure 5a), the kidneys were broad, bean-shaped, shiny, and reddish-brown in color. No changes in the organization of the glomeruli and renal tubules were observed. The renal tissue structures were intact and well organized, the glomeruli showed normal size, the renal tubules did not depict any signs of swelling or degeneration, and the interstitial regions did not exhibit inflammatory cell infiltration or fibrosis. The renal tubular epithelial cells were normal in size and the distal tubules did not show any detectable signs of expansion. A few transparent tubules in the renal tubules were observed, and no significant inflammatory cell infiltration in the stroma was detected. In the model group, glomerular hypertrophy, renal interstitium edema and a mass of inflammatory cell infiltration and fibrosis revealed in the interstitial regions were researched. The prednisone acetate and tripterygium glycoside tablet treatment groups illustrated that the glomerular size was slightly larger, and there existed a spot of inflammatory cell infiltration. The kidneys of the periostracum cicadae group showed a significant increase in size, dark red in color, with a small amount of inflammatory cell infiltration. 

In the untreated control group (Figure 5b), no proliferative glomerular basilar membrane, mesangium, or glomerular sclerosis was observed. In the model group, hyperplasia of glomerular mesenterium and basilar membrane tubular, swelling accompanied by segmental glomerulosclerosis, collapsed glomerular vascular loops, and perivascular inflammatory cell infiltration, were observed. The prednisone acetate and tripterygium glycoside tablet treatment groups showed proliferative glomerular mesangium and basilar membrane were inconspicuous, with minimal inflammatory cell infiltration. The periostracum cicadae treatment group exhibited mesangial thickening, but was better than the model group, as well as a small number of inflammatory cell infiltration.

### 2.5. Periostracum Cicadae Induces Cellular Apoptosis in the IgAN Rat Model

To gain insights on whether periostracum cicadae induces apoptosis in vivo, paraffin sections from the Sprague-Dawley (SD) rats were subjected to H&E staining (Figure 5a). The increase in the number of TdT-mediated dUTP nick end-labeling (TUNEL)-positive cells clearly demonstrates that cellular apoptosis was detected (Figure 5c). The microscopic signs of cellular apoptosis showed that cellular shrinkage, condensed, bright eosinophilic cytoplasm, and pyknotic small, dark nuclei were demonstrated, due to chromatin condensation. Figure 5a,c showed no chromatin condensation and normal cell size in the untreated control group. In the model group, chromatin condensation and apoptosis of glomerular epithelial cells were examined. The prednisone acetate and tripterygium glycoside tablet treatment groups indicated apoptosis of inflammatory cells were rare. The result of coloration selecting TUNEL in the periostracum cicadae treatment group clearly demonstrates that periostracum cicadae induces inflammatory cellular apoptosis in vivo.

### 2.6. The Effect of Periostracum Cicadae on TGF-β1- and Fas-Positive Cells

**Figure 6:**
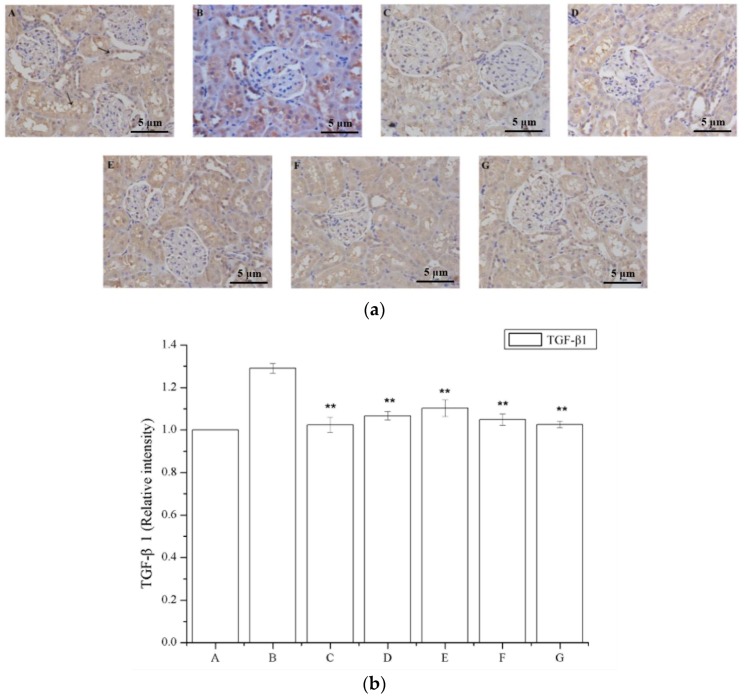

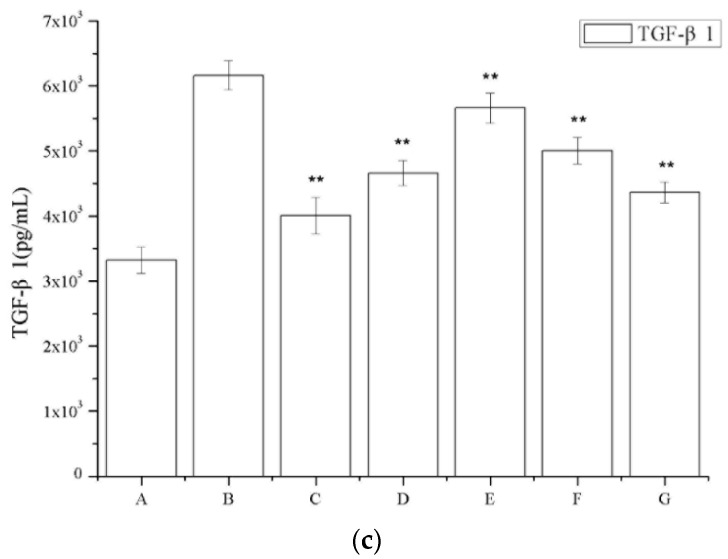
Effects of periostracum cicadae on the levels of TGF-β1 in the kidney. (**a**) Representative immunohistochemistry image. A: Normal, healthy control group; B: IgAN model group; C: prednisone acetate group; D: tripterygium glycoside tablet group; E–G: treatment group that received 0.5, 1, and 2 g/kg periostracum cicadae; (**b**) the quantitative analysis of TGF-β1 protein in kidney tissues. Data represent the mean ± standard deviation (** *p* < 0.01 vs model group); (**c**) effects of periostracum cicadae on the levels of TGF-β1 in the tissue of IgAN rats (** *p* < 0.01).

Figure 6a shows that a region of the glomeruli positively immunostained for TGF-β1, whereas this was not observed in the saline-treated group; a larger TGF-β1 immunostained area was observed in the model group (Figure 6a), and a smaller area was detected in the periostracum cicadae treatment group using doses of 0.5, 1.0, and 2.0 g/kg/day. The prednisone acetate and tripterygium glycoside tablet treatment groups showed a significant increase in the TGF-β1-positive area within the glomeruli. Immunohistochemical staining of kidney sections showed that prednisone acetate significantly decreased the secretion of TGF-β1 in rats with IgAN (*p* < 0.05, *p* < 0.01 vs IgAN model group; Figure 6b). Consistent with the immunohistochemical staining results, ELISA also revealed a suppressive effect of periostracum cicadae on TGF-β1 expression (Figure 6c).

**Figure 7:**
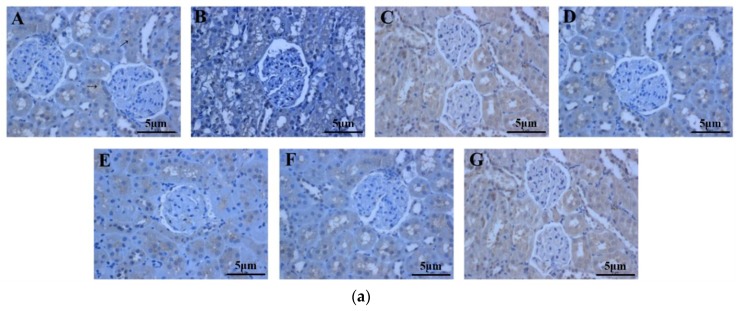

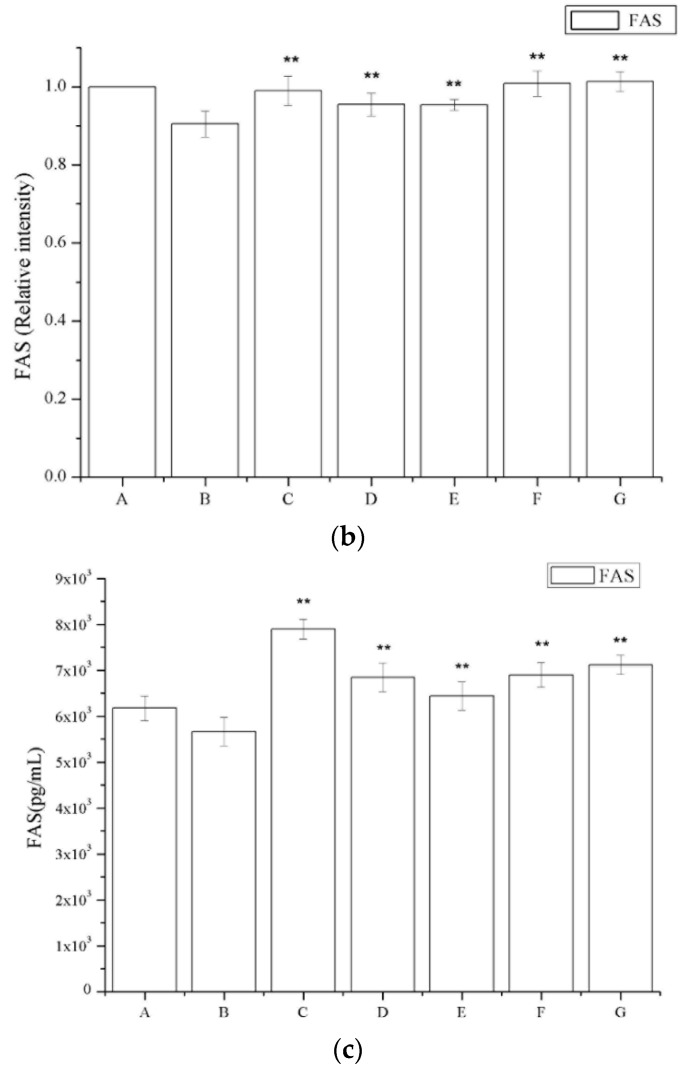
Effects of periostracum cicadae on FAS levels in the kidney. (**a**) representative immunohistochemistry image A: normal, healthy control group; B: IgAN model group; C: prednisone acetate group; D: tripterygium glycoside tablet group; E–G: treatment group that received 0.5, 1, and 2 g/kg periostracum cicadae; (**b**) the quantitative analysis of Fas protein in kidney tissues. Data represent the mean ± standard deviation (** *p* < 0.01 vs model group); (**c**) effects of periostracum cicadae on the levels of FAS in the tissue of IgAN rats (** *p* < 0.01).

The number of Fas-positive cells in the IgAN rats was lower compared to that in the saline-treated group (Figure 7a). The periostracum cicadae treatment group showed an increase in the number of Fas-positive cells. Figure 7b shows that prednisone acetate induces a marked increase in FAS expression (*p* < 0.01), whereas that in the IgAN group was significantly lower than in the normal, healthy control group, and increased in a dose-dependent manner after periostracum cicadae administration. The FAS levels determined by ELISA in the model group were lower compared to the untreated control group, whereas the levels were significantly induced in the treatment group relative to that in the model group (Figure 7c).

### 2.7. Assessment of TLR4, MCP-1, IgA, and Caspase 3 Expression Levels

**Figure 8:**
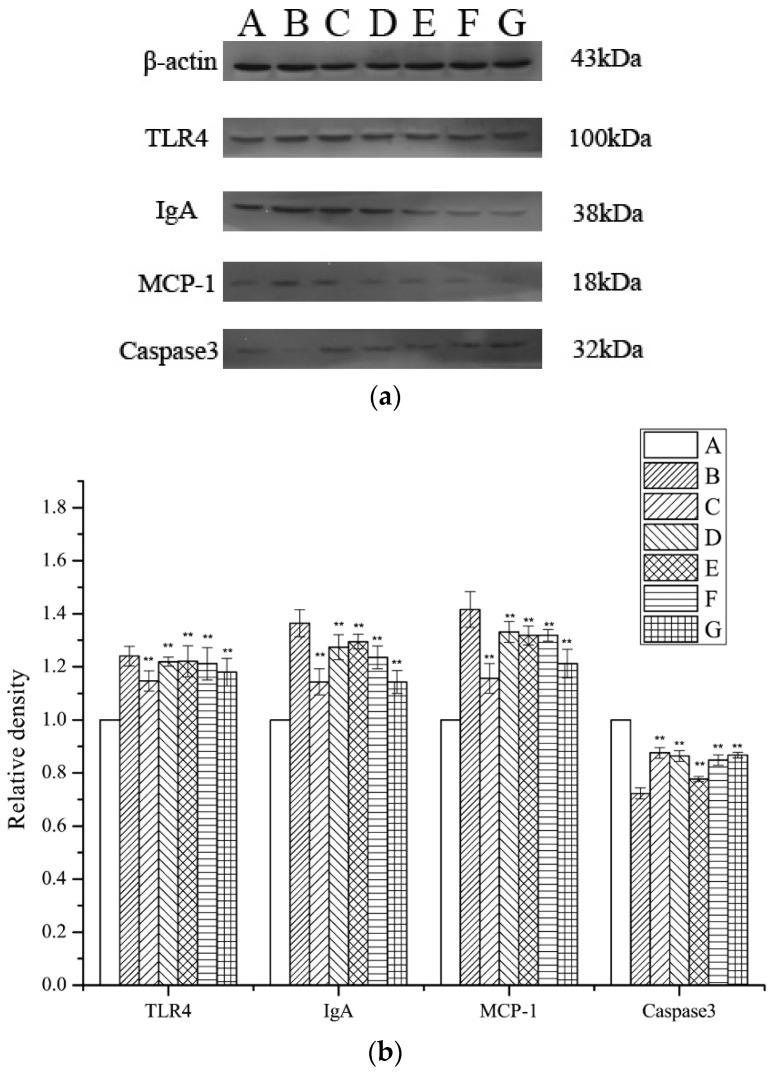
TLR4, MCP-1, IgA, and caspase-3 expression in rat kidney tissues. (**a**) TLR4, IgA, MCP-1, and caspase-3 expression levels were analyzed by Western blotting. (**b**) Quantitative analysis of TLR4, IgA, MCP-1, and caspase-3 protein levels. A: Healthy, normal controls; B: IgAN model group; C: prednisone acetate group; D: tripterygium glycoside tablet group; E–G: treatment group that received 0.5, 1, and 2 g/kg periostracum cicadae. Data represent the mean ± standard deviation (** *p* < 0.01 vs model group).

The levels of Monocyte Chemotactic Protein 1 (MCP-1), Toll-like receptor 4 (TLR4), IgA, and caspase 3 expression were investigated by Western blotting, using β-actin for normalization. A significant inhibition of TLR4, MCP-1, and IgA expression in the periostracum cicadae-treated rats was observed relative to that in the model group. The expression of TLR4, MCP-1, and IgA was significantly lower in the periostracum cicadae-treated rats than that in the model group. Figure 8 shows that the normal, healthy controls exhibited lower TLR4, MCP-1, and IgA expression levels, whereas these were significantly higher in the IgAN groups. However, after periostracum cicadae treatment, TLR4, MCP-1, and IgA expression gradually decreased. These findings suggest that periostracum cicadae treatment mitigates MCP-1, TLR4, and IgA expression in the IgAN rats. The untreated control group exhibited a relatively higher caspase 3 expression level than the IgAN groups. However, after periostracum cicadae treatment, caspase 3 expression gradually increased. Caspase 3 activity is an integral step in most apoptotic events. In the present study, treatment with periostracum cicadae resulted in the upregulation of caspase 3. These results show that periostracum cicadae plays a key role in inducing apoptosis in IgAN rats.

## 3. Discussion

IgA nephropathy is characterized by increased levels of galactose-deficient IgA1 (Gd-IgA1) that is complicated by nephritis, and Gd-IgA1 is now believed to play a pivotal role in the pathogenesis of both IgA nephropathy. In addition, IgA-containing complexes have been found in the glomeruli of IgA nephropathy and IgAN patients. IgA nephropathy can damage the liver, and M. Zaidi et al., reported hepatitis in association with IgAN, thus far, have been in conjunction with viral infections secondary to hepatitis A, B, and C [23]. Research indicated ALT and AST levels significantly increased in the IgAN model group. In the present study, we found that endocapillary hypercellularity, tubular atrophy, and interstitial fibrosis negatively affect renal survival. Furthermore, chronic lesions, such as tubulointerstitial fibrosis, have been associated with poor prognosis in patients with IgAN [24,25]. To date, the pathogenesis of IgAN, which often leads to sclerosis of the glomeruli, remains unclear. Previous studies have suggested that proinflammatory cytokines, such as IL-1β, IL-2, IL-4, IL-5, IL-6, IL-8, and TNF-α, may play an aggravating role in the development of inflammation and glomerular damage in IgAN [26,27,28,29].

Toll-like receptors (TLRs) are key components of the mammalian innate immune system. TLRs are known to be involved in the pathogenesis of various inflammatory diseases, including kidney diseases, such as ischemic acute kidney injury, organ transplant rejection, and immune-mediated glomerulonephritis [30]. In the kidneys, TLR4 is expressed primarily in the proximal and distal tubules and in Bowman’s capsular epithelia [31,32,33,34,35], whereas minimal expression occurs in the glomeruli and endothelial cells [36]. Under stress or inflammatory conditions, the expression of TLR4 is upregulated in various regions of the nephron [31,37]. Previous studies have shown that TLR4-mediated upregulation of IL-6, IL-1β, and MCP-1 is significantly correlated with TLR4 and MCP-1 expression. MCP-1 plays a central role in inflammation, and causes tubulointerstitial lesions by recruiting target cells, which include macrophages, monocytes, T cells, neutrophils, eosinophils, and basophils, into the tubulointerstitium, where the target cells themselves secrete cytokines, such as TGF-β1 and TNF-α [38,39]. These have also shown that renal TLR4 expression is closely associated with MCP-1, TGF-β1, and IL-6 expression, thereby suggesting that TLR4 plays a key role in chronic renal pathophysiology. Patients with severe proteinuria show upregulated renal TLR4 expression, thereby suggesting aggravation of inflammatory and fibrotic processes. Except for the inflammatory response, oxidative stress causes the formation of reactive oxygen species, leading to renal injury [40]. TGF-β1 contributes to the development of tubulointerstitial fibrosis [41,42]. Furthermore, TGF-β1 secreted by mesangial and inflammatory cells in this model is closely involved in inflammatory reactions and the accumulation of extracellular matrix [43,44]. Increased expression of the TGF-β1 gene is associated with glomerulosclerosis [45] and increased severity of tubulointerstitial damage. The results of the present study show that high and low doses of periostracum cicadae can dramatically decrease protein expression in the blood and urine in the rat models. Furthermore, high doses of periostracum cicadae were determined to be more effective than low doses. Significant IgA deposition in the glomeruli was also observed in these animals. These findings indicate the successful establishment of an IgAN rat model. The results of the present study show a significant decrease in serum IgA, TNF-α, IL-1β, and IL-6 levels, which in turn, suppress the growth of glomerular mesangial cells and their extracellular matrix, improve IgAN symptoms, and protect renal function. Immunohistochemical analysis indicated a larger TGF-β1-immunostained area in the glomeruli of the IgAN models (Figure 6), which was significantly decreased after treatment with periostracum cicadae at doses of 0.5, 1.0, and 2.0 g/kg/day. TLR4, IgA, and MCP-1 were downregulated in the periostracum cicadae-treated rats compared to that in the IgAN animal models. In conclusion, the present study has shown that the administration of periostracum cicadae improves renal tissue inflammation and fibrotic processes.

Apoptosis of renal glomerular cells is known to contribute to the regulation of renal cell proliferation, as well as induce the repair of damaged renal tissues. The degree of glomerular cell apoptosis is commensurate with the decrease in the number of glomerular cells, accumulation in the extracellular matrix, mesangial cell proliferation, and proteinuria in the progression of glomerulosclerosis [46]. Fas, which is also known as apoptosis antigen-1 or cluster of differentiation 95, is a member of the death receptor family, a subfamily of the tumor necrosis factor receptor superfamily [47]. Interactions between Fas and its natural ligand (Fas L) or agonistic antibodies induce apoptosis in responsive cells [47]. Caspase-3 is a member of the cysteine-aspartic acid protease (caspase)/IL-1β-converting enzyme family [48], and is directly activated by caspase 8, 9, and 10 via extrinsic and intrinsic pathways that initiate apoptosis. Previous studies have revealed that caspase 3 expression is positively associated with Fas and Fas L expression in human cells [49,50]. The present study demonstrated that caspase 3 expression is correlated with Fas expression, similar to that described in previous reports [49,50,51]. The present study also determined that Fas and caspase 3 are upregulated in renal tissues, as indicated by TUNEL staining, and are associated with glomerular injury. Furthermore, the number of Fas-positive cells and the expression of proteins involved in the caspase 3 pathway in IgAN rats decreased relative to that in the saline-treated group. However, the periostracum cicadae-treatment group showed an increase in the number of Fas-positive cells and the levels of caspase 3 expression. Therefore, increased Fas and caspase 3 expression promoted apoptosis in IgAN rat cells.

In summary, the pathological evaluation of kidney tissues indicates that rats treated with the high doses of periostracum cicadae exhibit less kidney inflammation and fibrosis than the IgAN rats. We also determined that the administration of periostracum cicadae significantly reduces TLR4, TGF-β1, MCP-1, and IgA expression in the IgAN rats. The results of the present study also demonstrate that the increase in the expression of Fas and its downstream signaling molecules, such as caspase 3, may alleviate the symptoms of IgAN in patients.

## 4. Materials and Methods

### 4.1. Reagents

Enzyme-linked immunosorbent assay (ELISA) kits for rat TNF-α (Shanghai Xitang, Shanghai, China), IL-6 (Shanghai Xitang), and IL-1β (Shanghai Xitang) were used according to the manufacturer’s instructions. Rabbit anti-TLR4 polyclonal antibody (Boster Biological Technology, Pleasanton, CA, USA), rabbit anti-IgA polyclonal antibody (Boster Biological Technology), rabbit anti-MCP-1 polyclonal antibody (Boster Biological Technology), and horseradish peroxidase (HRP)-labeled goat anti-rabbit IgG antibody (Boster Biological Technology) were used in the detection of the corresponding target proteins. Radioimmunoprecipitation assay (RIPA) lysis buffer (Solarbio Biological Technology, Beijing, China), bicinchoninic acid (BCA) protein assay kit (Solarbio Biological Technology), horseradish peroxidase (HRP)-conjugated secondary antibodies (Boster Biological Technology), and an enhanced chemiluminescent (ECL) detection kit (Beyotime, Haimen, China) were also procured.

### 4.2. Preparation of Prednisone Acetate Extract

Dried periostracum cicadae was purchased from a traditional medicine market in Xinjiang, China and identified by Professor Jincai Lu of the School of Traditional Chinese Materia Medica of Shenyang Pharmaceutical University. A voucher specimen (No. 20081001) was deposited in the Research Department of Natural Medicine of Shenyang Pharmaceutical University. Dried periostracum cicadae (5 kg) was refluxed three times with 70% ethanol (50 L) to obtain a crude extract.

### 4.3. Animals

One hundred and forty male SD rats (average weight: 140 ± 20 g) were obtained from the Animal Center of Urumqi (Certificate No. SCXK (xin) 2011-0003, Urumqi, China). The SD rats were kept and fed in a clean-grade room at a constant temperature (18 °C) and humidity (45%) with a 12 h light/dark cycle. Routine urine testing was performed after one week of pre-feeding using the test strip method. Rats that showed no hematuria or proteinuria were selected and randomly distributed to each group. All animal experiments were performed under institutionally approved protocols and complied with the Guide for the Care and Use of Laboratory Animals (Approval Letter of Animal Experimental Ethical Inspection of First Affiliated Hospital, Shihezi University School of Medicine, Approval Number A2017-154-01, 5 January 2017).

### 4.4. Establishment of a IgAN Model

The IgAN animal model, established based on the IgA nephropathy model and gavage of hot drugs, was used to establish blood stasis and heat syndrome. The rats were randomly divided into the following seven groups: untreated controls, IgAN model (IgAN group), prednisone acetate control (PAG group, 6.3 mg/kg), tripterygium glycoside tablet control (9.4 mg/kg), and three groups received oral periostracum cicadae (0.5, 1, and 2 g/kg). The rat IgAN model was established by administering bovine serum albumin (BSA), lipopolysaccharide (LPS), and carbon tetrachloride (CCl_4_) as previously described (Table 2) [52,53,54], with slight modifications. Briefly, the immunogen BSA was intragastrically administered at a dose of 4 mL/kg once every two days for eight consecutive weeks, and 0.1 ml of CCl_4_ in 0.3 mL of castor oil was given once weekly for nine weeks. On the sixth week of treatment, 0.05 mg of LPS (Sigma Chemical Co., St. Louis, MO, USA) was injected through the tail vein once every two weeks, for another six weeks, to establish blood stasis and heat syndrome, symptoms that characterize the IgAN model. On the ninth week, 25% ginger ale (table 2) was intragastrically administered at a dose of 10 mL/kg once every two days for 4 consecutive weeks. For the untreated control group, 4 mL/kg distilled water was used instead of BSA, 0.4 mL of castor oil was employed to replace castor oil and CCl_4_, and saline was used instead of LPS on the 6th week. The administration methods were the same as that in the model group.

### 4.5. Sample Collection and Preparation

At the end of 16 weeks of treatment, 24 h urine samples were collected with metabolic cages, and the model was identified based on the levels of 24 h urine protein, BUN, and serum creatinine (Scr). Blood samples were obtained from the abdominal aorta upon sacrifice, and were used in measuring ALT, AST, TP, TCHO, TAG, BUN, and CREA, using an automatic biochemistry analyzer.

In addition, rats from each group were sacrificed at different time points, and their kidneys were collected for histological examination. Each kidney was divided into three portions. The first portion was fixed in 40 g/L formaldehyde, embedded in paraffin, cut into sections, and then stained with eosin (H&E) and periodic acid-Schiff (PAS) for light microscopy. The second portion was embedded in optimal cutting temperature compound (OCT) and stored at −70 °C, followed by frozen sectioning for immunofluorescence staining. The third portion was stored at −70 °C for Western blotting.

### 4.6. Biochemical Analysis of Serum Samples

The blood samples were centrifuged at 1500 rpm for 10 min at 4 °C to isolate the serum, which was stored immediately at −80 °C. Serum IgA, TNF-α, IL-1β, and IL-6 levels were determined using commercially available ELISA kits, according to the manufacturer’s instructions. The optical density value was determined using a microplate reader (Thermo Varioskan Flash 3001, Waltham, MA. USA) and calculated at the linear portion of the curve. The experiment was repeated thrice under the same conditions.

### 4.7. Histopathological Examination

After blood collection, the kidneys were fixed in 10% (*v*/*v*) neutral formalin for 24 h. To enhance fixation, the kidneys were placed in a rubber-capped ampulla bottle that was 2/3 filled with 10% (*v*/*v*) neutral formalin, and then vacuumed to allow complete immersion of the tissues. The kidney tissues were paraffin-embedded, sectioned (Leica, Nussloch, Germany) at a thickness of 5 μm, and stained with H&E for subsequent cell infiltration observation. Alternatively, sections were stained with PAS to examine mucus production. Quantitative analysis of mucus production was performed using Image-Pro Plus 6.0 (Media Cybernetics, Bethesda, MD, USA).

### 4.8. Immunofluorescence

The 5 μm thick frozen kidney sections were mounted on glass slides and air-dried. The tissue sections were stained with fluorescein isothiocyanate (FITC)-labelled polyclonal rabbit anti-IgA (diluted 1:10). IgA deposition was graded using a five-stage semi-quantitative method as follows: (−), no staining at low magnification and possible staining at high magnification; (+), possible staining at low magnification and staining at high magnification; (++), staining at low magnification and distinct staining at high magnification; (+++) distinct staining at low magnification and more intense staining at high magnification; and (++++), very intense staining at high magnification. In this study, most of the staining in the IgAN mode group ranged from ++ to +++.

### 4.9. TUNEL Assay

The TUNEL staining kit was purchased from Roche (Basel, Switzerland). The staining procedure was as follows. (1) The tissue sections were deparaffinized by immersing the slides in xylene and rehydrated by sequentially immersing the slides across an ethanol gradient. (2) Endogenous peroxidase activity was quenched by adding 0.3% H_2_O_2_ for 10 min, and then washed in 10 mmol/L PBS (pH 7.4) thrice for 5 min each. (3) The sections were digested with 20 μg/mL proteinase K for 25 min at room temperature and then washed in 10 mmol/L PBS (pH 7.4) thrice for 5 min each time. (4) The TUNEL reaction mixture was added to each section, followed by incubating in a humid chamber for 60 min at 37 °C. (5) Then, the slides were washed in 10 mmol/L PBS (pH 7.4) thrice for 5 min each and incubated with peroxidase conjugated antibody in a humid chamber for 30 min at 37 °C. The slides were then washed in 10 mmol/L PBS (pH 7.4) thrice for 5 min each time. (6) The tissue sections were stained with DAB for 5 min, counterstained with hematoxylin, dehydrated, cleared in xylene, and covered with a glass slip.

### 4.10. Immunohistochemical Staining

The paraffin-embedded tissue sections were dewaxed, hydrated, treated with 5 mmol/L levamisole to block endogenous alkaline phosphatase, and incubated with blocking serum for 30 min at room temperature to reduce nonspecific background staining. The sections were rehydrated in phosphate-buffered saline/0.1% BSA for 15 min, before addition of the appropriate blocking serum for an additional 15 min. The sections were incubated with polyclonal rabbit anti-TGF-β1 (dilution of 1:1000; Chemicon, Temecula, CA, USA) and rabbit anti-FAS 15 g/mL (Santa Cruz Biotechnology, Santa Cruz, CA, USA) overnight at 4 °C. The following day, the slides were rinsed, incubated with biotinylated goat anti-rabbit IgG (Abcam, Massachusetts, UK) and then processed using an alkaline phosphatase-streptavidin-biotin immunoperoxidase method (Beijing Zhongshan Biotechnology Co., Ltd., Beijing, China). The tissue sections were counterstained with hematoxylin. Negative controls for specific labeling were performed in parallel by replacing the primary antibody with normal rabbit serum. Renal cortex sections were digitally imaged and quantitatively examined using a computer-assisted image analysis software (CX41 light microscopy; Olympus, Tokyo, Japan). To quantitate the amount of chymase-positive TGF-β1 and the area of FAS-positive expression in the tubulointerstitial compartment, 50 fields consecutively selected in the cortical areas of the kidneys were examined at a magnification of 400×. Fields containing glomeruli and large arteries were excluded. To quantitate the expression of TGF-β1 and FAS in the tubulointerstitial compartment, the average percentage of positively staining proportional area and the whole area was calculated by image analysis.

### 4.11. Western Blot Analysis

The nuclear and cytoplasmic proteins were extracted from the kidney tissues using an NE-PER kit (Pierce Biotechnology, Rockford, IL, USA) according to the manufacturer’s instructions. The proteins were separated by sodium dodecyl sulfate-polyacrylamide gel electrophoresis and transferred onto a polyvinylidene fluoride (PVDF) membrane (Millipore Corp., Billerica, MA, USA), which was then incubated with an anti-TLR4 antibody (Cell Signaling Technology, Lexington, KY, USA), an anti-IgA antibody (Cell Signaling Technology), an anti-caspase 3 antibody (Cell Signaling Technology), an anti-MCP-1 antibody (Cell Signaling Technology), and anti-β-actin antibody (Cell Signaling Technology) at 4 °C overnight. After washing, the membrane was incubated with a horseradish peroxidase-conjugated secondary antibody for 1 h. After washing five times with TBST, the membranes were incubated with an HRP-conjugated antibody for 1 h at room temperature. Western blots were developed using ECL (Thermo, Lithuania, EU) and were exposed on Kodak radiographic film.

### 4.12. Statistical Analysis

Data were expressed as the mean ± SD of at least three independent experiments and evaluated using ANOVA, followed by Bonferroni correction. *p* < 0.05 was considered statistically significant. The analyses were performed using SPSS 17.0 statistical software (IBM, Chicago, IL, USA) program.

## Figures and Tables

**Table 1:** IgA fluorescence intensities.

Group	Controls	IgAN Model Group	Prednisone Acetate Group	Tripterygium Glycoside Tablet Group	Periostracum Cicadae Group		
					0.5 g/kg	1 g/kg	2 g/kg
IgA deposits	−	++++	+	++	+++	++	+

**Table 2:** Induction of IgAN in the SD rats.

Control	BSA Was Replaced by Distilled Water; PBS Was Used as Injection Control	
IgAN	BSA, 4 mL/kg	Intragastrically every 2 days for 8 weeks
	CCL_4_/castor oil (25%, *v*/*v*)	subcutaneous injection (S.C.), 0.4 mL, once a week for 9 weeks
	LPS, 0.05 mg	Caudal iv, once every two weeks for six weeks at the 6th week
	25% ginger ale, 10 mL/kg	Once every two days for four consecutive weeks on the 9th week
